# *In Silico* Prediction of Quercetin Analogs for Targeting Death-Associated Protein Kinase 1 (DAPK1) Against Alzheimer’s Disease

**DOI:** 10.2174/1570159X22666240515090434

**Published:** 2024-05-15

**Authors:** Yilu Sun, Jia Zhao, Yizhu Lu, Fung Yin Ngo, Bo Shuai, Zhang-Jin Zhang, Yibin Feng, Jianhui Rong

**Affiliations:** 1 Department of Chinese Medicine, The University of Hong Kong Shenzhen Hospital, Shenzhen, China;; 2 School of Chinese Medicine, The University of Hong Kong, 3 Sassoon Road, Pokfulam, Hong Kong, China;; 3 Zhu Nansun’s Workstation and Yu Jin’s Workstation, School of Chinese Medicine, The University of Hong Kong, 3 Sassoon Road, Pokfulam, Hong Kong, China;; 4 Department of Integrated Traditional Chinese and Western Medicine, Union Hospital, Tongji Medical College, Huazhong University of Science and Technology, Wuhan, China

**Keywords:** *In silico* prediction, quercetin, quercetin analogs, death-associated protein kinase 1 (DAPK1), Alzheimer’s disease (AD), neurodegenerative disease

## Abstract

Alzheimer’s Disease (AD) is a progressive neurodegenerative disorder that greatly affects the health and life quality of the elderly population. Existing drugs mainly alleviate symptoms but fail to halt disease progression, underscoring the urgent need for the development of novel drugs. Based on the neuroprotective effects of flavonoid quercetin in AD, this study was designed to identify potential AD-related targets for quercetin and perform *in silico* prediction of promising analogs for the treatment of AD. Database mining suggested death-associated protein kinase 1 (DAPK1) as the most promising AD-related target for quercetin among seven protein candidates. To achieve better biological effects for the treatment of AD, we devised a series of quercetin analogs as ligands for DAPK1, and molecular docking analyses, absorption, distribution, metabolism, and excretion (ADME) predictions, as well as molecular dynamics (MD) simulations, were performed. The energy for drug-protein interaction was predicted and ranked. As a result, quercetin-A1a and quercetin-A1a1 out of 19 quercetin analogs exhibited the lowest interaction energy for binding to DAPK1 than quercetin, and they had similar dynamics performance with quercetin. In addition, quercetin-A1a and quercetin-A1a1 were predicted to have better water solubility. Thus, quercetin-A1a and quercetin-A1a1 could be promising agents for the treatment of AD. Our findings paved the way for further experimental studies and the development of novel drugs.

## 1 INTRODUCTION

Alzheimer’s Disease (AD) is a progressive neurodegenerative disorder that affects a large number of the elderly population and constitutes a heavy financial burden on society [1]. AD emerged as the sixth leading cause of death in the United States in 2020, affecting more than 5 million citizens, while the prevalence may reach 14 million by 2050, highlighting a major burden for the healthcare system [2]. The pathology of AD is characterized by senile plaques and neurofibrillary tangles, which are known as the results of the extracellular deposition of misfolded β-amyloid protein (Aβ) and the intracellular hyperphosphorylation of tau proteins,respectively [3, 4]. In the clinic, AD progressively displays various symptoms, such as memory loss and cognitive decline [5]. AD patients are initially affected by executive function deficits and episodic memory difficulties, while proteolytic product Aβ is undetectable [6]. With disease progression over time, Aβ peptide accumulates in the neocortex and is detectable in the cerebrospinal fluid of dementia patients [7].

Aβ peptide is well known to induce apoptosis, promote oxidative stress, and increase neurotoxin production through different mechanisms involving apoptosis signal-regulating kinase, redox factor-1, and several other signaling proteins [8-11]. Accumulation of neurofibrillary tangles also leads to neuronal death [12]. Apart from the standalone effects, hyperphosphorylated tau may interact with Aβ peptide to exaggerate the pathological changes in the brain [13, 14]. Consequently, Aβ and tau protein could synergistically disrupt synaptic plasticity and promote neuroinflammation, thereby hallmarking the pathology of AD [15-17]. DAPK1 is a calcium/calmodulin-dependent serine/threonine kinase [18]. Overexpressed DAPK1 augmented tau phosphorylation and promote neuronal death [19]. DAPK1 was elevated in the brains of AD patients, and it can cause AD by Aβ production and tau hyperphosphorylation [20]. Activation of DAPK1 could result in memory loss, and inhibition of DAPK1 could attenuate memory impairment in mice [21]. Cyclin-dependent kinase 1 (CDK1) mRNA and protein expression could be increased by Aβ oligomers in AD [22]. Polo-like kinase 1 (PLK1) accelerated Aβ-induced neuron death in AD [23]. Insulin-like growth factor 1 receptor (IGF1R) was increased in the AD patients [24]. Tyrosine-protein kinase Met (c-MET) was reduced in hippocampal pyramidal neurons, and the decline of c-MET caused neuron damage in AD [25]. Increased Epidermal growth factor receptor (EGFR) may be related to Aβ-induced memory loss in AD [26]. Dopamine receptor D4 (DRD4) single nucleotide polymorphism was related to AD [27]. Enormous efforts have been made to develop new drugs for targeting different pathological processes, including Aβ, tau protein, neuroinflammation, oxidative stress, cell cycle, and neurotransmitter receptors [28-33]. However, current anti-AD drugs mainly improve the symptoms through inhibiting cholinesterase (*e.g*., donepezil, galantamine, rivastigmine) or non-competitively antagonizing NMDA receptor signaling (*e.g*., memantine), while none of these drugs could reverse the cognitive impairment [34, 35]. Trehalose, solanezumab, and crenezumab are known to decrease Aβ generation and boost Aβ clearance [36-38]. Inhibition of tau oligomerization and aggregation is also evaluated as another primary therapeutic strategy [39, 40]. Interaction with the vitagene system to prevent oxidative stress was reported as a therapeutic strategy for neurodegenerative disorders [41, 42]. Moreover, anti-inflammatory drugs appear to be effective in alleviating symptoms of AD, although the clinical efficacy remains controversial [43]. Nevertheless, current treatments could prevent the progression of AD and improve cognitive function at an early stage but failed to reverse severe symptoms of patients with late-stage AD [44]. Thus, there is an urgent need for the development of novel therapeutics to target the complex pathological changes in AD.

Long-term oxidative stress and inflammatory insults are well-known to drive the formation of Abeta plaques and tau neurofibrillary tangles, leading to age-related decrements in neuronal function and behavioral manifestations [45, 46]. The etiology of AD is complex; therefore, multitarget compounds have been explored from natural products to treat AD [47]. Phytochemical compounds, typically alkaloids, flavonoids, and triterpenes, have proven efficacy in targeting multiple pathological processes, such as Aβ aggregation, neuroinflammation, oxidative stress and insulin resistance [48, 49]. Quercetin is a typical flavonoid with two benzene rings joined together by a heterocyclic ring with a phenolic group, as shown in Fig. (**1A**), and is ubiquitously produced in vegetables, fruits, and tea [50, 51]. Quercetin is well-known for its antioxidative, anti-cancer, antiviral and neuroprotective effects [52-54]. Consequently, quercetin is clinically evaluated for treating diabetes, cancer, kidney disease, cardiovascular disease, arthritis and severe acute respiratory syndrome [55-62]. In the context of AD, quercetin exhibited neuroprotective properties by improving memory recall, learning performance, and cognitive function in AD patients or aged AD mice [63, 64]. The underlying molecular mechanisms may involve the inhibition of cell death, suppression of proinflammatory mediator NF-B, and induction of antioxidant enzyme heme oxygenase-1 [62, 65]. Furthermore, quercetin readily scavenges reactive radicals by donating hydrogen from its phenolic group to the radicals, protecting neurons from oxidative damage and cell death [62, 66, 67]. DAPK1 was elevated in AD patients, and quercetin reduced the DAPK1 expression in Aβ1-42 treated HT-22 cells [68]. It showed promising neuroprotection in AD [69, 70]. Thus, we hypothesized that quercetin could be a potential lead compound for the development of effective anti-AD treatments. Although quercetin showed promising neuroprotection in AD, its low bioavailability limits the clinical application of quercetin [69, 70]. Interestingly, chemical modifications may result in various quercetin analogs with better antioxidant and anti-inflammatory effects [71]. Molecular docking and molecular dynamics simulations can be applied to study the molecular interaction of quercetin and its analogs with targets, and quantitative structure-activity relationship can be used to analyze their bioavailability to predict potential anti-AD drugs [72]. In this study, we performed network pharmacology analysis and molecular docking to identify potent molecular targets for quercetin. By targeting specific AD-related proteins, we designed and virtually screened a series of modified quercetin analogs for the development of quercetin-based anti-AD drugs.

## 2 AD-RELATED PROTEIN TARGETS SCREENING

**Fig. (1):**
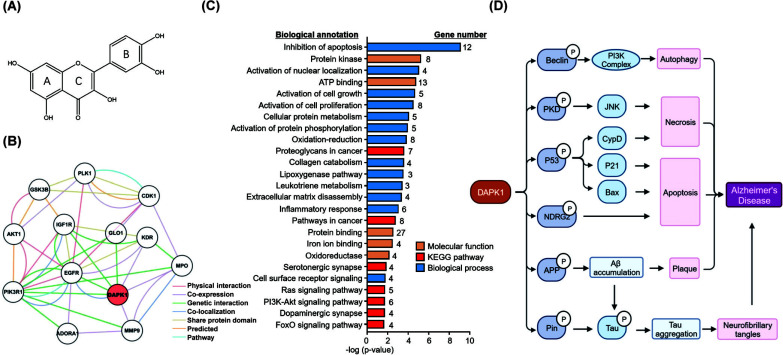
(**A**) Chemical structure of quercetin. (**B**) Protein-protein interaction network of DAPK1 and targets in “inhibition of apoptosis.” ADORA1, Adenosine A1 Receptor; AKT1, Protein kinase B alpha; CDK1, Cyclin-dependent kinase 1; DAPK1, Death-associated protein kinase 1; EGFR, Epidermal Growth Factor Receptor; GLO1, Glyoxalase 1; GSK3B, Glycogen synthase kinase-3 beta; IGF1R, Insulin-Like Growth Factor 1 Receptor; KDR, Vascular endothelial growth factor receptor 2; MMP9, Matrix Metallopeptidase 9; MPO, Myeloperoxidase; PIK3R1, Phosphatidyl inositol 3 kinase; PLK1, Polo-like kinase 1. (**C**) Biological and functional characterization of the protein targets. (**D**) The role of DAPK1 in Alzheimer’s Disease. DAPK1, Death-associated protein kinase 1; PKD, protein kinase D; NDRG2, NDRG Family Member 2; APP, Amyloid Beta Precursor Protein; PIK3R1, Phosphatidyl inositol 3 kinase; JNK, c-Jun N-terminal kinase; CypD, Cyclophilin D; Aβ, Amyloid beta.

To identify AD-related biological targets for quercetin, we initiated the virtual screening with the SwissDock algorithm, examined the gene-disease association with DisGeNET and subsequently analyzed the Gene ontology (GO) and the Kyoto Encyclopaedia of Genes and Genomes (KEGG) enrichment annotations [73, 74]. As a result, 68 proteins were first predicted as potential protein targets for quercetin in SwissDock, while 38 AD-related proteins were identified in DisGeNET (Table **S1**). The AD-related candidates for quercetin were further verified by searching medical literature. GO and KEGG pathway enrichment analyses were performed using DAVID 6.8 (http://david.ncifcrf.gov), with annotations having an adjusted *p* < 0.05 considered significantly enriched (Fig. **1C**). As a result, “inhibition of apoptosis” (GO:00433066) was found as the most significant GO term under “biological process,” whereas “protein kinase activity” (GO:0004672) was the most significant GO term under “molecular function.” Consistently, enrichment analysis also found several annotations related to cell survival and proliferation, including “activation of cell growth” (GO:0030307) and “activation of cell proliferation” (GO:0008284). Pathway enrichment analysis identified 26 KEGG pathways, including “proteoglycans in cancer” (hsa05205), “pathways in cancer” (hsa05200), “serotonergic synapse” (hsa04726), “Ras signaling pathway” (hsa04014), “PI3K-Akt signaling pathway” (hsa04151), “dopaminergic synapse” (hsa04728) and “FoxO signaling pathway” (hsa04068). Moreover, GO and KEGG enrichment analysis selected seven potential AD-related proteins, namely death-associated protein kinase 1 (DAPK1), cyclin-dependent kinase 1 (CDK1), polo-like kinase (PLK1), insulin-like growth factor 1 (IGF1R), tyrosine-protein kinase Met (c-MET), epidermal growth factor receptor (EGFR) and dopamine receptor D4 (DRD4).

## 3 PROTEIN-PROTEIN INTERACTION (PPI) NETWORK CONSTRUCTION

To further explore the biological function of potential targets and analyze the interactions between them, we constructed a PPI network with GeneMANIA (http://www.genemania.org), focusing on targets from the top GO term “inhibition of apoptosis” (GO:00433066). The targets were imported into the GeneMANIA system, and “Homo sapiens” was selected as the search organism. The PPI network figure was developed with Cytoscape 3.10.0. As illustrated in Fig. (**1B**), DAPK1 had genetic interactions with PIK3R1, EGFR, IGF1R, and GLO1 [75]. It co-localized with EGFR and co-expressed with KDR (VEGFR2), MPO, and MMP9 [76-78].

## 4 MOLECULAR DOCKING

To simulate the interactions between quercetin and seven AD-related protein targets, we docked quercetin into the crystal structures of these proteins with Autodock Vina software. Molecular docking was conducted according to the previously described methodology [79]. For protein preparation, the three-dimensional crystal structures of various proteins were downloaded from the Protein Data Bank (https://www.rcsb.org/), and the identifiers were listed in Table **1**. The protein structures were imported into PyMOL (https://www.pymol.org/2/), where water molecules and ligands were removed from the protein structures. Polar hydrogens and charges were added, and the location was optimized by UCSF Chimera (https://www.cgl.ucsf.edu/chimera/). For ligand preparation, the three-dimensional structure of quercetin was obtained from ChemDraw and Chem3D. To evaluate the structure-activity relationship, 19 quercetin analogs were also designed for screening. The chemical structures of quercetin analogs were constructed and converted to three-dimensional structures by ChemDraw and Chem3D. Energy minimization was performed using the MM2 force field in Chem3D. Subsequently, the proteins and ligands were subjected to molecular docking analyses using AutoDock Vina (http://vina.scripps.edu/) in PyRx (https://pyrx.sourceforge.io/). The interaction energies were predicted and ranked. The drug-protein interactions were visualized by PyMOL and LigPlot (https://www.ebi.ac.uk/thornton-srv/software/LigPlus/). As shown in Table **1**, quercetin binds to DAPK1, DRD4, and PLK1 with interaction energy of -9.3,-9.1, and -8.7 kcal/mol, respectively, representing the top three strong binding partners. Considering that quercetin showed the highest affinity towards DAPK1, DAPK1 might be a promising target for quercetin. Therefore, we selected DAPK1 as the target protein for further virtual study of the structure-affinity relationship. DAPK1 could positively mediate tumor necrosis factor-alpha (TNF-α) and gamma-interferon (IFN-γ) induced programmed cell death [80]. It is mainly expressed in the cortical neurons of the aged human frontal cortex [81]. The overexpression of DAPK1 is also related to Aβ accumulation and tau dysregulation in AD [82, 83]. It could cause Aβ accumulation by triggering the phosphorylation of Amyloid Precursor Protein (APP) and further accelerating the hyperphosphorylation of tau [84, 85]. DAPK1 can promote neuronal cell death through autophagy, necrosis, and apoptosis, and it may contribute to the loss of neurons in AD. In addition, DAPK1 activation led to memory loss and spatial learning decline in AD mice, while the inhibition rescued synaptic loss and improved memory and spatial learning [86]. Previous *in vitro* studies showed that quercetin inhibited DAPK1 mRNA expression against Aβ challenge [68]. Interestingly, melatonin not only decreased DAPK1 expression in a post-transcriptional manner in neuronal cell lines and mouse primary cortical neurons but also directly bound to DAPK1 and facilitated the ubiquitination for proteasome-mediated degradation [87]. Taken together, these studies confirmed the role of DAPK1 in the pathogenesis and progression of AD. Thus, quercetin may have a therapeutic potential for the treatment of AD *via* targeting DAPK1.

**Fig. (2):**
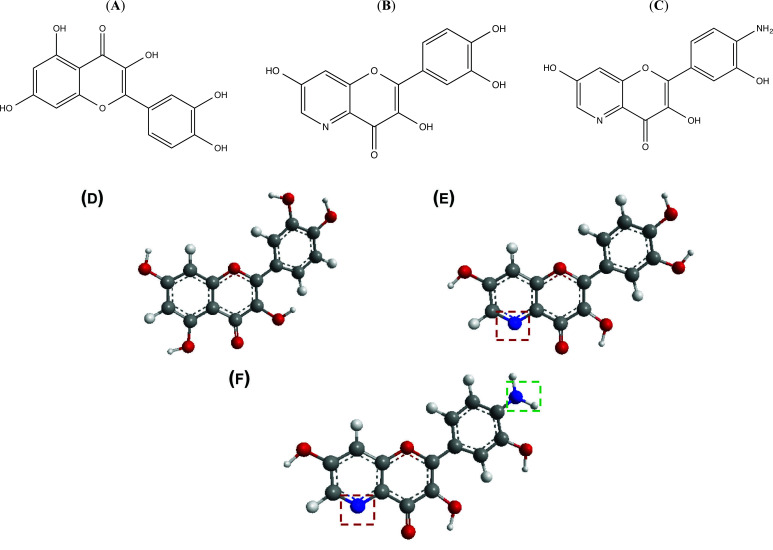
(**A**) 2D structure of quercetin; (**B**) 2D structure of quercetin-A1a; (**C**) 2D structure of quercetin-A1a1; (**D**) 3D structure of quercetin; (**E**) 3D structure of quercetin-A1a; (**F**) 3D structure of quercetin-A1a1. Modified sites were marked with red and green squares.

**Fig. (3):**
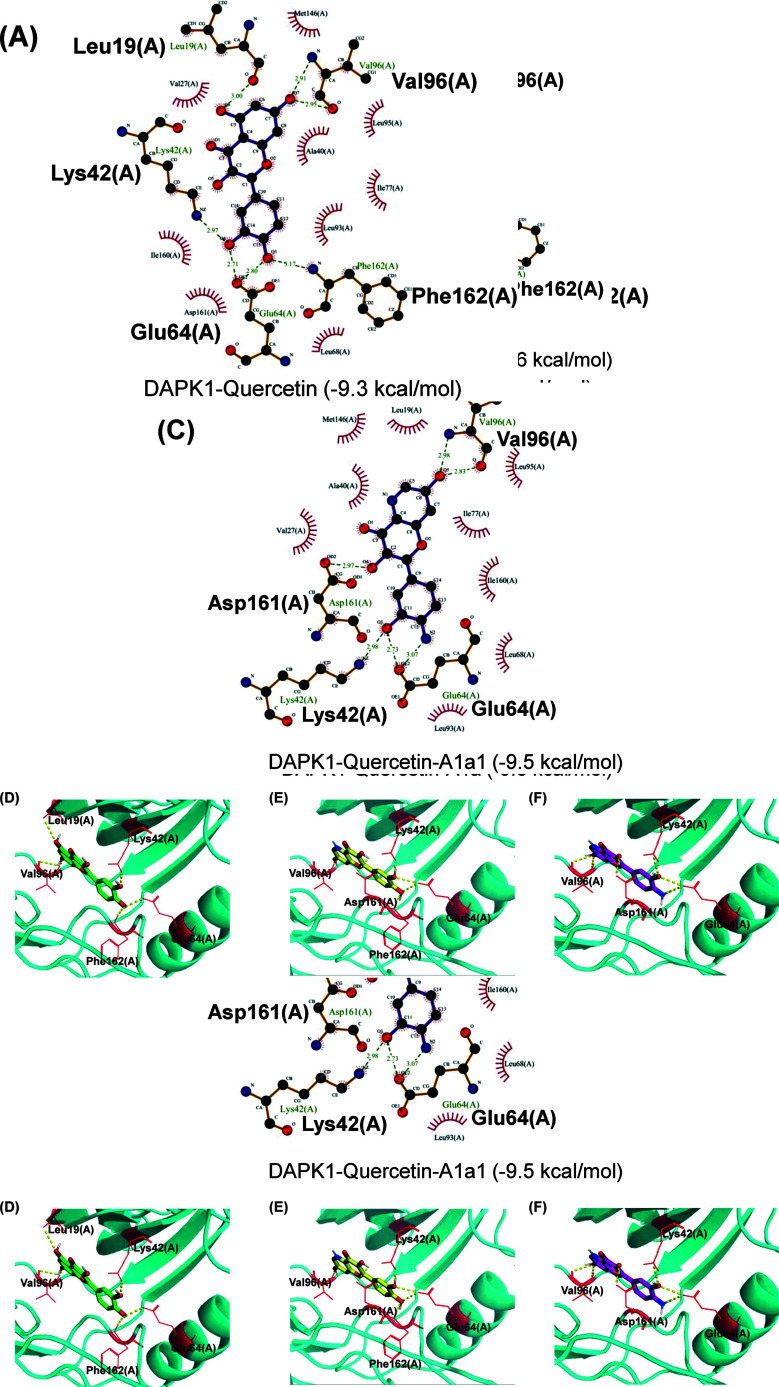
Molecular docking analyses of DAPK1 with the top three compounds. (**A**) 2D predicted interaction between quercetin and DAPK1; (**B**) 2D predicted interaction between quercetin-A1a and DAPK1; (**C**) 2D predicted interaction between quercetin-A1a1 and DAPK1; (**D**) 3D predicted interaction between quercetin and DAPK1; (**E**) 3D predicted interaction between quercetin-A1a and DAPK1; (**F**) 3D predicted interaction between quercetin-A1a1 and DAPK1.

The therapeutic application of quercetin is limited by its low bioavailability and water solubility [88]. Chemical modification of the quercetin scaffold could yield an array of analogs, while some displayed potentially better anticancer properties for clinical applications [89]. We attempted to introduce specific chemical moieties to enhance the pharmacokinetic properties, such as water solubility, blood-brain barrier (BBB) permeability, and bioavailability. The nitrogen atoms in the forms of the amino, N-dimethylamino, and pyridinyl substitutions could introduce positive charges, which potentially enhance the binding of the compounds to endothelial cells in the BBB for better disposition of these compounds in the brain. The positive charges could enhance electrostatic interaction with target proteins. On the other hand, the carboxylic group was chosen to increase the polarity and water solubility of these compounds. Furthermore, the polar groups could form hydrogen bonds with the target proteins for higher affinity and better specificity. Therefore, we designed 19 quercetin analogs by introducing four different chemical moieties, namely, amino, N-dimethylamino, carboxylic and pyridinyl substitutions, to various positions in the quercetin structure (Tables **2** and **3**). These quercetin analogs were analyzed by docking to the structure of DAPK1. The binding of each quercetin analog was ranked in Table **3** by the interaction energy. Quercetin-A1a, Quercetin-A1a1, and quercetin were the three strongest compounds for binding to DAPK1. Quercetin-A1a was generated from quercetin by replacing the A ring with hydroxypyridine (Figs. **2A**, **2B**, **2C**, **2D**, **2E** and **2F**). Kaempferol and morin were the natural analogs of quercetin and alleviated the memory deficit in AD [90, 91]. The interaction energy between kaempferol, morin and quercetin and DAPK1 were -8.2, -7.1 and -9.3 kcal/mol, respectively (Table **3**). Quercetin-A1a is bound to DAPK1 with an interaction energy of -9.6 kcal/mol, whereas quercetin is bound to DAPK1 with an interaction energy of -9.3 kcal/mol (Table **4**). The replacement at the C-5 position of quercetin yielded the analog quercetin-A1a with increased interaction affinity (Table **4** and Fig. **3**). The introduction of pyridinyl may increase the hydrogen bonding force and ionic effects, leading to changes in amino acid positioning. The replacement of the C-5 phenolic hydroxyl with other chemical groups at the C-5, conversely, the products like quercetin-A1 are bound to DAPK1 with considerably decreased affinity compared with the parent quercetin. We further rearranged the hydroxyl groups on the benzene ring of quercetin-A1a from ortho- to meta-arrangement, yielding another analog termed morin-A1a (Table **3**). The molecular docking revealed that morin-A1a is bound to DAPK1 with the interaction energy of -8.0 kcal/mol (Table **3**). Morin-A1a is bound to DAPK1 with decreased binding affinity compared with quercetin-A1a and quercetin. Such structure-activity relationship basically agreed with drug design through the re-positioning of chemical modifiers, suggesting a crucial role in drug-protein interaction [92]. By examining the involvement of different amino acid residues, we found that quercetin-A1a, quercetin-A1a1 and quercetin mainly interacted with common residues, including Lys 42(A), Glu 64(A) and Val 96 (A) in DAPK1 (Fig. **3**). To investigate the cross-interactions of quercetin, quercetin-A1a, and quercetin-A1a1 with other AD-related targets, we performed similar molecular docking of these three compounds into the structures of DAPK1, CDK1, PLK1, IGF1R, c-MET, EGFR, DRD4. The interaction energy was calculated and ranked as shown in Tables **1** and **4**. Quercetin-A1a, quercetin-A1a1 and quercetin displayed the strongest interactions with DAPK1 relative to other AD-related proteins, while quercetin-A1 exhibited the highest affinity to all seven protein targets. These results suggested that DAPK1 appeared to be the most favorable target for three quercetin compounds compared with other protein targets. Quercetin-A1a and quercetin-A1a1 might be potential novel lead compounds for targeting DAPK1 in the treatment of AD.

## 5 ADME PREDICTION

**Fig. (4):**
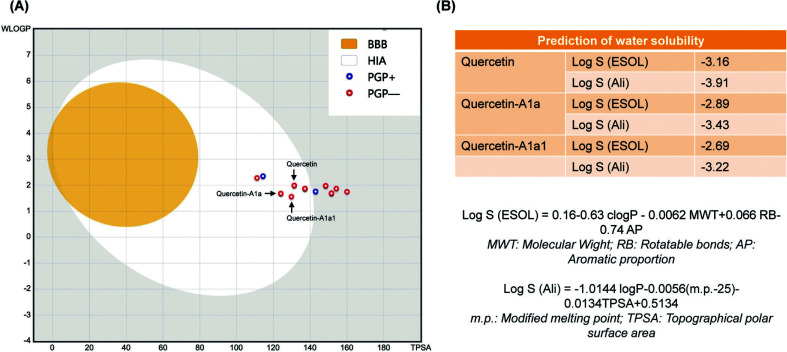
(**A**) BOILED egg Model prediction; (**B**) Water solubility prediction (Solubility class: Log S scale Insoluble < -10 < Poorly < -6 < Moderately < -4 < Soluble < -2 Very < 0 < Highly).

The ADME prediction study of quercetin and its 19 analogs was performed by using the SwissADME (http://www.swissadme.ch/). The predictions for passive human gastrointestinal absorption (HIA) and blood-brain barrier (BBB) permeation were demonstrated with the BOILED-Egg model (Fig. **4**). Quercetin-A1a, quercetin-A1a1 and quercetin were predicted to show good gastrointestinal absorption (in the white area) and were identified as non-substrate for permeability glycoprotein (red dots). Remarkably, quercetin-A1a and quercetin-A1a1 showed lower TPSA and WLOGP scores and better gastrointestinal absorption capacity compared with quercetin [93]. Water solubility (Log S) of quercetin, quercetin-A1a and quercetin-A1a1 was predicted with ESOL and Ali methods [94, 95]. Quercetin-A1a and quercetin-A1a1 had higher Log S (ESOL) and Log S (Ali) compared with quercetin. These results suggested that pyridyl and amino groups were introduced to quercetin, and its water solubility could be improved.

## 6 MOLECULAR DYNAMICS SIMULATION

Molecular dynamics (MD) simulations were carried out for the three strongest compounds for binding to DAPK1, quercetin-A1a, quercetin-A1a1 or quercetin complexing with DAPK1 in MOE (Molecular Operating Environment, 2019) using the Nosé-Poincaré Andersen (NPA) equations of motion [96]. Before MD calculations, MMFF94x was selected as a force field, droplet water in a sphere shape with six margins was added as a solvent, and energy minimization was performed to optimize the system. With a timestep of 0.002 ps, coordinates and parameters were stored every 0.5 ps. The MD calculations were run under default protocols for 600 ps (equilibrium for 100 ps and production for 500 ps) [97]. The system pressure *versus* time (P-t) graphic and total system energy *versus* time (E-t) graphic were shown in Fig. (**5**). Both of the analogs would not change the stability of the protein. Quercetin-A1a and quercetin-A1a1 showed similar dynamic performance with quercetin, and quercetin-A1a showed higher energy compared with quercetin-A1a1. Both quercetin-A1a and quercetin-A1a1 had the potential to be used to treat AD *via* targeting DAPK1.

## CONCLUSION

AD is a multifactorial neurodegenerative disorder characterized by progressive cognitive decline and memory loss [98]. Despite extensive research efforts, no cure and even effective medicines are currently available for AD [99]. The existing treatments for AD primarily focus on symptomatic relief and do not halt the disease progression [100]. DAPK1 presents an opportunity for developing new disease-modifying treatments [101].

Natural products are well-known to serve as structural scaffolds for chemical modifications or rational design in drug discovery [102, 103]. Quercetin protected primary neurons by attenuating Aβ-induced cytotoxicity and improved cognitive and memory function in the mouse model of AD [62, 104]. However, the low bioavailability is the key factor to limit the clinical application of quercetin. We used *in silico* approaches to identify potential quercetin analogs for the treatment of Alzheimer's Disease. The present study identified DAPK1 as the most promising target for quercetin through bioinformatic analyses. Chemical modification yielded quercetin-A1a and quercetin-A1a1 with enhanced binding affinity towards DAPK1. According to MD simulation and ADME prediction, we expect that quercetin-A1a and quercetin-A1a1 may be promising anti-AD treatment *via* targeting DAPK1.

## Figures and Tables

**Fig. (5):**
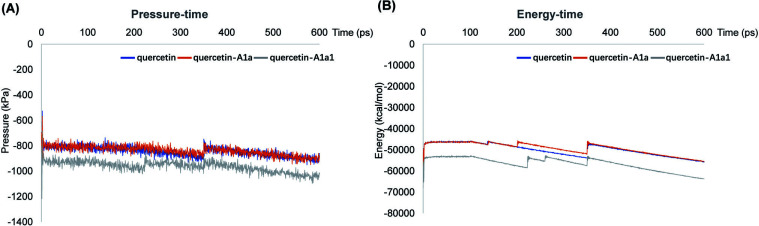
MD simulation. (**A**) The evaluation of potential system pressure of quercetin, quercetin1A1a and quercetin-A1a1 with DAPK1 as a function of time. (**B**) The evaluation of potential system energy of quercetin, quercetin1A1a and quercetin-A1a1 with DAPK1 as a function of time.

**Table 1:** The interaction energy between quercetin and AD-related candidate proteins.

**Symbol**	**Description**	**PDB ID**	**Energy (kcal/mol)**
DAPK1	Death-associated protein kinase 1	5AUZ	-9.3
CDK1	Cyclin-dependent kinase 1	4YC6	-8.0
PLK1	Polo-like kinase 1	2YAC	-8.7
IGF1R	Insulin-like growth factor 1	5FXR	-8.2
c-MET	Tyrosine-protein kinase Met	5YA5	-7.6
EGFR	Epidermal growth factor receptor	6LUB	-7.3
DRD4	Dopamine receptor D4	5WIU	-9.1

**Table 2:** Types of chemical modifications in quercetin analogs.

**Substituent**	**Name**
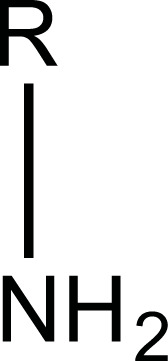	Amino
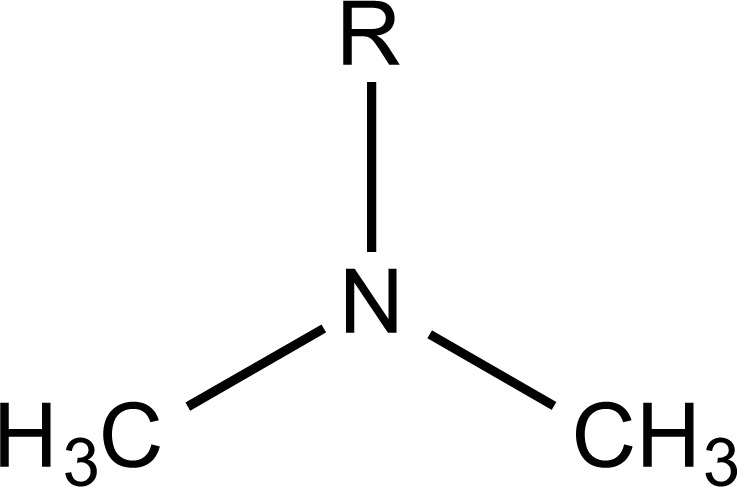	N-dimethylamino
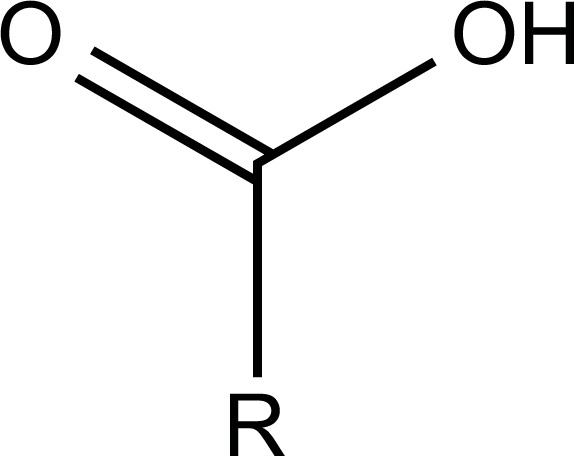	Carboxylic
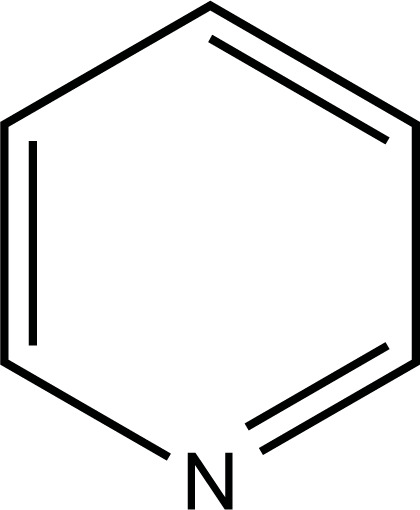	Pyridinyl

**Table 3:** Chemical structure of 19 quercetin analogs and interaction energy between quercetin analogs and DAPK1.

**Compounds**	**Chemical Structure**	**Interaction Energy (kcal/mol)**
Quercetin-A1 (Q-A1)	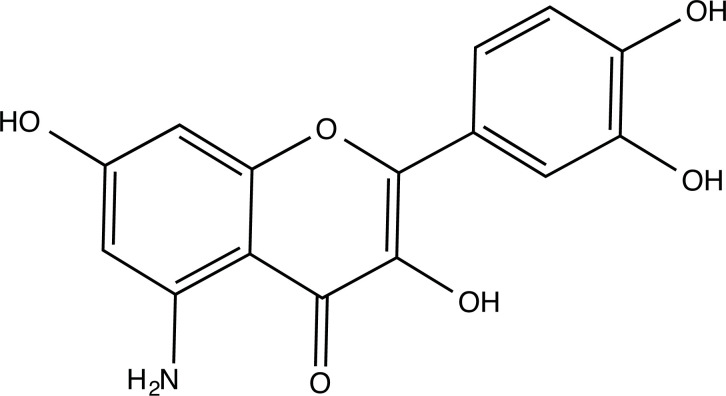	-7.2
Quercetin-A2 (Q-A2)	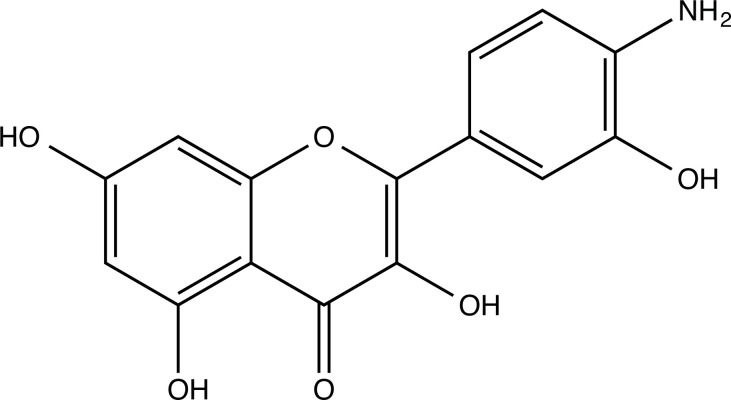	-8.0
Quercetin-A3 (Q-A3)	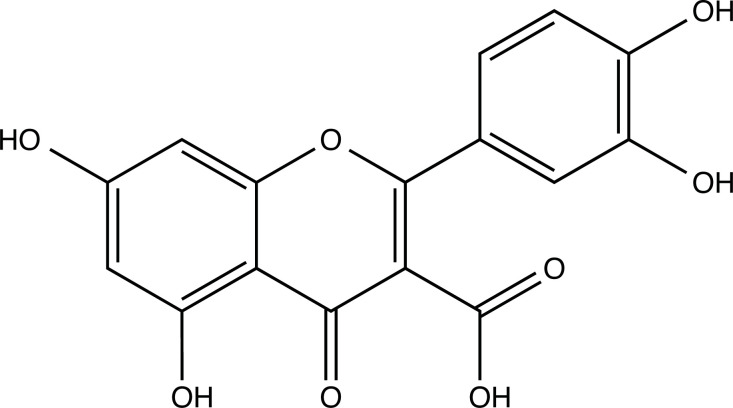	-7.5
Quercetin-A4 (Q-A4)	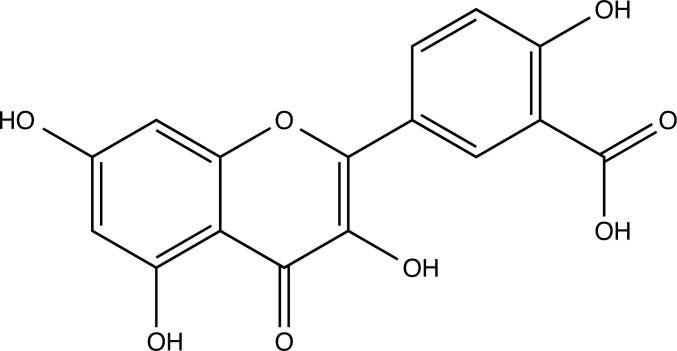	-8.6
Quercetin-A5 (Q-A5)	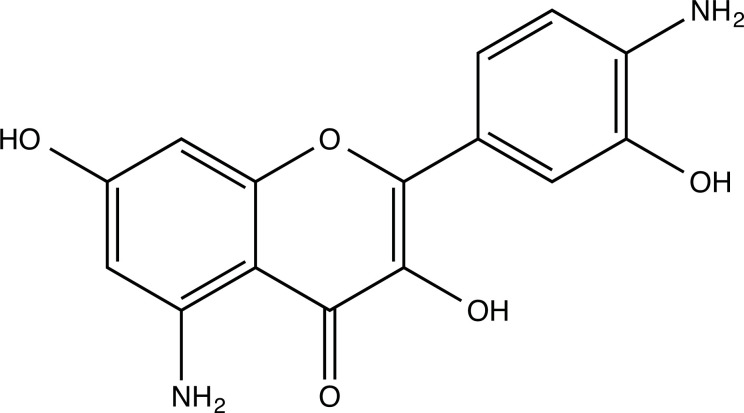	-8.1
Quercetin-A6 (Q-A6)	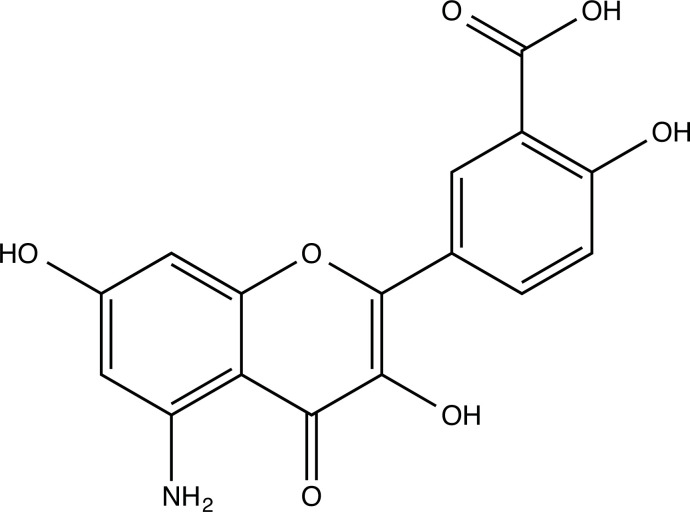	-8.4
Quercetin-A7 (Q-A7)	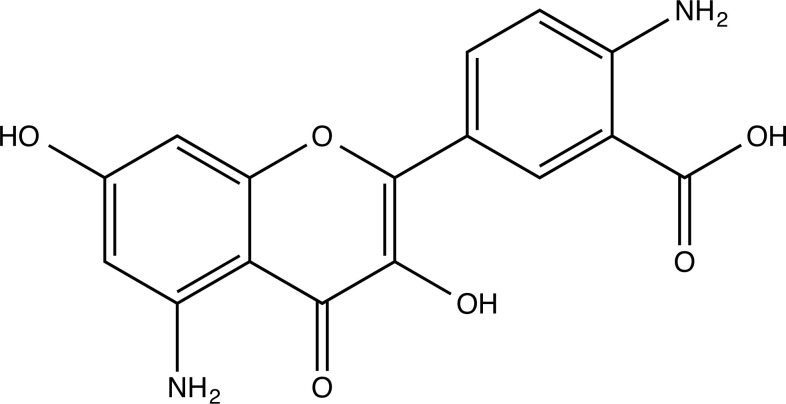	-8.5
Quercetin-A1a (Q-A1a)	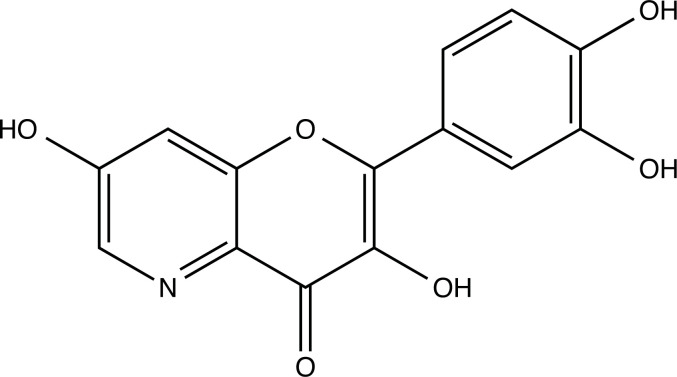	-9.6
Quercetin-A1a1 (Q-A1a1)	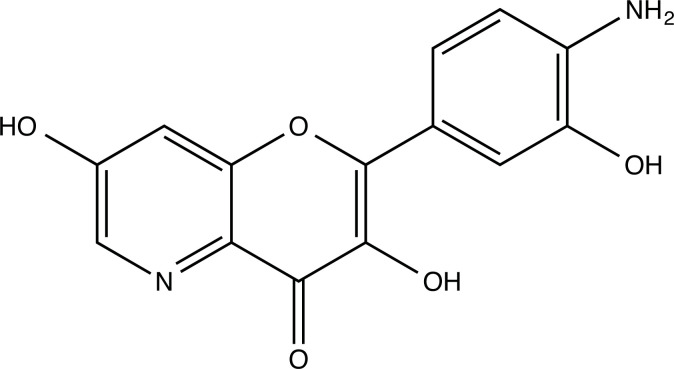	-9.5
Quercetin-A1b (Q-A1b)	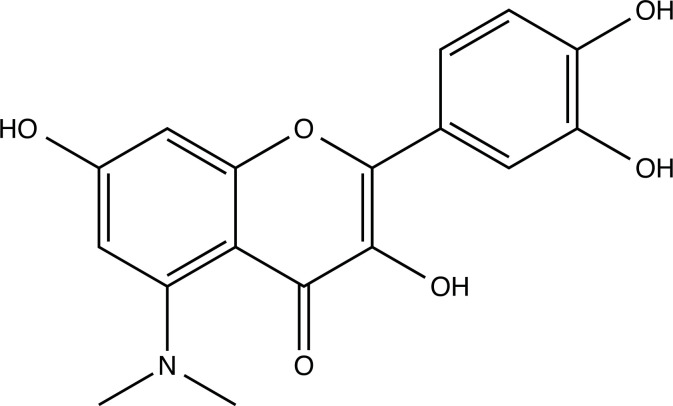	-7.2
Morin	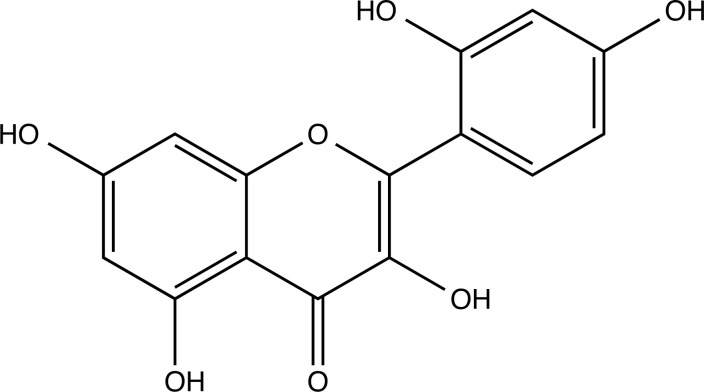	-7.1
Morin-A1 (M-A1)	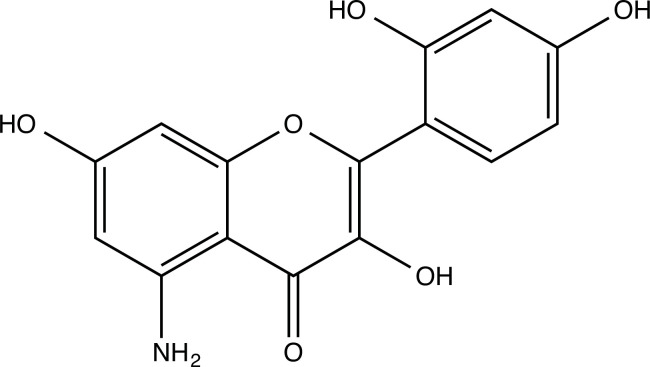	-7.2
Morin-A1a (M-A1a)	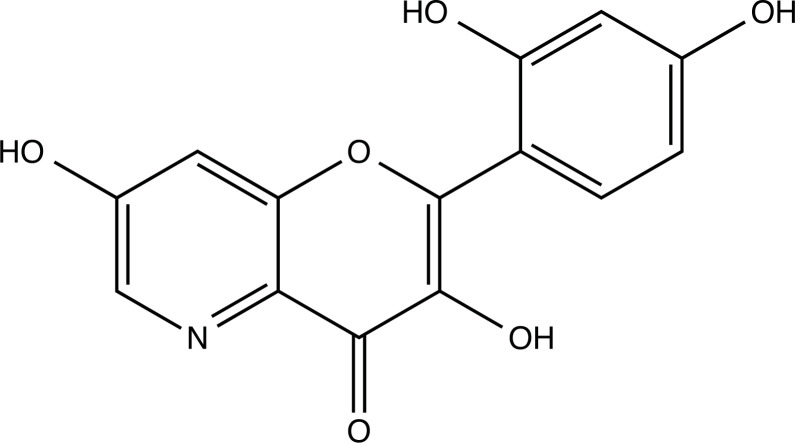	-8.0
Morin-A1b (M-A1b)	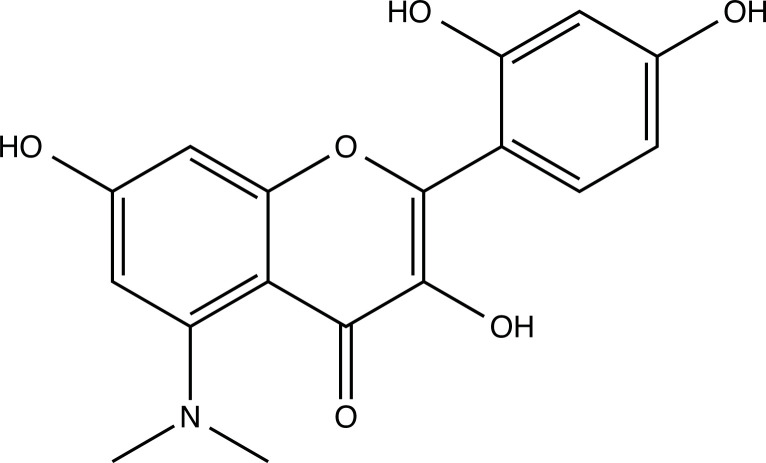	-7.2
Morin-A11 (M-A11)	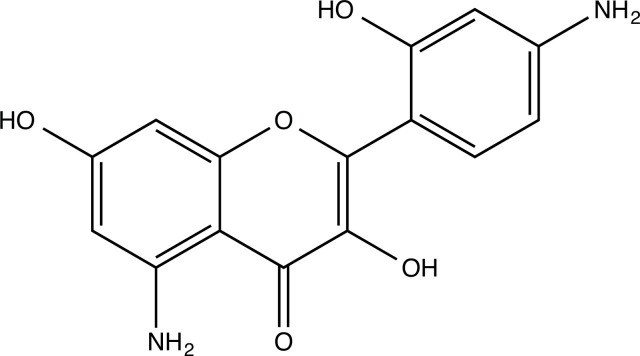	-9.3
Kaempferol	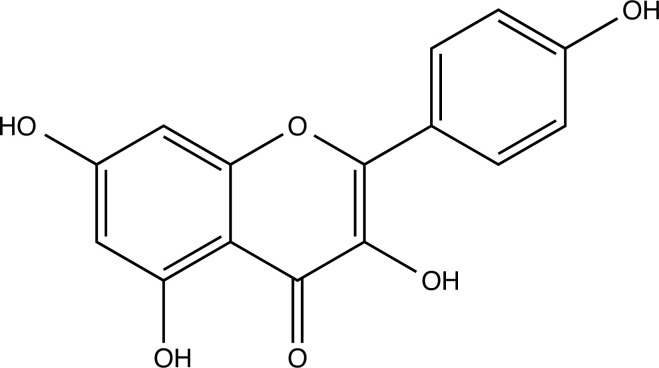	-8.2
Compound 1	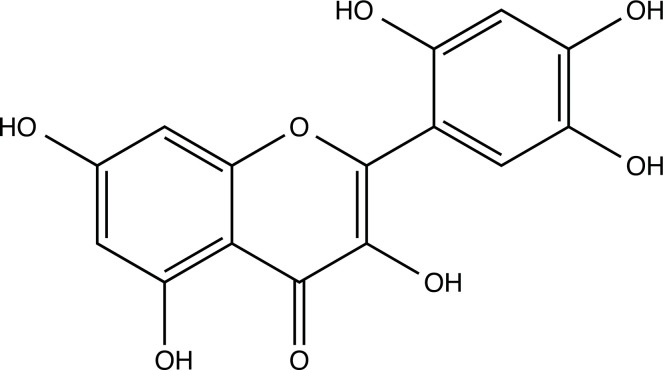	-7.5
Compound 2	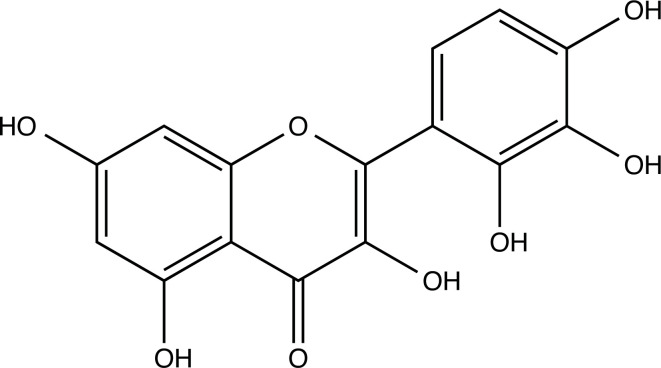	-7.1
Compound 3	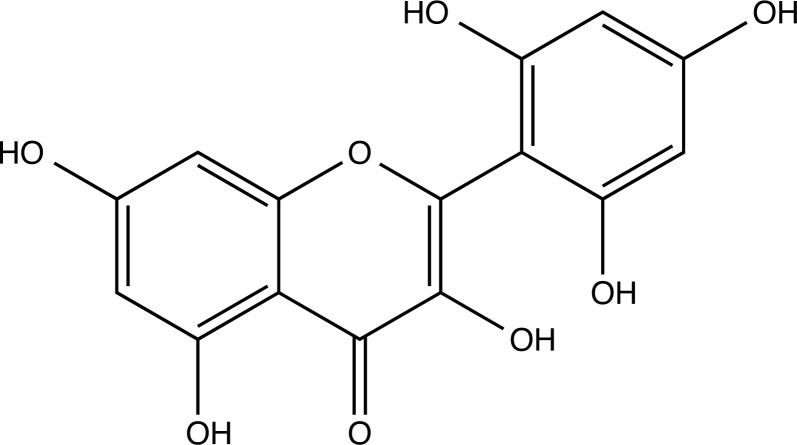	-6.9

**Table 4:** Interaction energy between quercetin-A1a or Quercetin-A1a1 and seven candidate proteins.

**Protein Name**	**Energy for Quercetin-A1a Binding (kcal/mol)**	**Energy for Quercetin-A1a1 Binding (kcal/mol)**
DAPK1	-9.6	-9.5
CDK1	-8.1	-7.7
DRD4	-8.5	-9.1
PLK1	-8.7	-8.0
IGF1R	-7.8	-7.4
EGFR	-7.5	-7.0
c-MET	-7.6	-7.6

## LIST OF ABBREVIATIONS

AD: Alzheimer’s Disease
BBB: Blood-brain Barrier
CDK1: Cyclin-dependent Kinase 1
c-MET: Tyrosine-protein Kinase Met
DAPK1: Death-associated Protein Kinase 1
EGFR: Epidermal Growth Factor Receptor
GO: Gene Ontology
HIA: Human Gastrointestinal Absorption
MD: Molecular Dynamics
PLK1: Polo-like Kinase 1
