# Supplementary material for: In Silico Prediction of Quercetin Analogs for Targeting Death-Associated Protein Kinase 1 (DAPK1) Against Alzheimer’s Disease
Source: Curr Neuropharmacol. 2024 May 15;22(14):2353–67. doi: 10.2174/1570159X22666240515090434 (PMC11451310; doi:10.2174/1570159X22666240515090434)
Supplement: Supplementary file 1 [file CN-22-2353_SD1.pdf]

Supplementary Material

In Silico Prediction of Quercetin Analogs for Targeting Death-Associated Protein Kinase 1 (DAPK1) Against Alzheimer's Disease

Yilu Sun[1,2,#], Jia Zhao[1,2,3,#], Yizhu Lu[2], Fung Yin Ngo[2], Bo Shuai[4], Zhang-Jin Zhang[1,2], Yibin Feng[2,*] and Jianhui Rong[2,*]

[1]*Department of Chinese Medicine, The University of Hong Kong Shenzhen Hospital, Shenzhen, China;* [2]*School of Chinese Medicine, The University of Hong Kong, 3 Sassoon Road, Pokfulam, Hong Kong, China;* [3]*Zhu Nansun's Workstation and Yu Jin's Workstation, School of Chinese Medicine, The University of Hong Kong, 3 Sassoon Road, Pokfulam, Hong Kong, China;* [4]*Department of Integrated Traditional Chinese and Western Medicine, Union Hospital, Tongji Medical College, Huazhong University of Science and Technology, Wuhan, China*

1875-6190/24 $65.00+.00 © 2024 Bentham Science Publishers

Table S1. List of AD-related protein targets for quercetin.

Symbol	Description	Role in AD	Disease Association Score	References
GSK3β	Glycogen synthase kinase-3 beta	• Tau hyperphosphorylation<br>• Aβ synthesis	0.7	[1]
BACE1	β secretase	• Aβ synthesis	0.6	[2]
IGF1R	Insulin-like growth factor-1 receptor	• Promote cellular metabolism<br>• Inhibit apoptosis	0.56	[3]
MPO	Myeloperoxidase	• Promote oxidative stress	0.5	[4]
PT	Prothrombin	• Tau proteolysis	0.33	[5]
ABCB1	P-glycoprotein	• Aβ clearance	0.1	[6]
AKR1C4	Aldo-keto reductase, family 1 member C4	• Bile acid synthesis	0.1	[7]
Akt1	Protein kinase B alpha	• Regulate mitochondrial pro-apoptotic mediators<br>• Suppress GSK3β	0.1	[8]
ALOX5	Arachidonate 5-lipoxygenase	• Fatty acid metabolism<br>• Resolve neuroinflammation	0.1	[9]
CDK1	Cyclin-dependent kinase 1	• Cell cycle re-entry	0.1	[10]
CYP19A1	Aromatase	• Cholesterol homeostasis<br>• Cognition	0.1	[11]
DAPK1	Death-Associated Protein Kinase 1	• Promote apoptosis and autophagy	0.1	[12]
MAOA	Monoamine oxidase A	• Metabolize neurotransmitters<br>• Promote oxidative stress	0.1	[13]
MMP9	Matrix metallopeptidase 9	• Aβ degradation	0.1	[14]
PLA2G1B	Secretory phospholipase A2	• Phospholipid metabolism<br>• Promote inflammation	0.1	[15, 16]
ABCG2	ATP-binding cassette G2 transporter	• Prevent Aβ influx<br>• Reduce oxidative stress	0.07	[17, 18]
MMP3	Matrix metalloproteinase 3	• Degrade nerve growth factor<br>• Cleave soluble Aβ	0.07	[19, 20]
EGFR	Epidermal growth factor receptor	• Promote Aβ aggregation	0.05	[21]
MMP2	Matrix metalloproteinase 2	• Cleave soluble Aβ<br>• Tau cleavage	0.05	[22]
ADORA2A	Adenosine A2a Receptor	• Decrease hippocampal neurogenesis	0.04	[23]
ALOX15	Arachidonate 15-lipoxygenase	• Fatty acid metabolism<br>• Resolve neuroinflammation	0.04	[24]
DRD4	Dopamine receptor D4	• Mesolimbic system neurotransmission	0.04	[25]
AKR1A1	Aldo-keto reductase, family 1 member A1	• Reduce oxidative stress	0.03	[26]
GLO1	Glyoxalase 1	• Reduce oxidative stress by detoxifying methylglyoxal	0.03	[27]
ABCC1	ATP-binding cassette C1 transporter	• Aβ clearance	0.02	[28]
CA2	Carbonic anhydrase 2	• Synaptic transformation	0.02	[29]

Symbol	Description	Role in AD	Disease Association Score	References
VEGFR2	Vascular endothelial growth factor receptor 2	• Vascular dysfunction	0.02	[30]
MET	Mesenchymal epithelial transition factor receptor	• Reduce neurogenesis<br>• Astrocyte activation	0.02	[31]
PLK1	Polo-like kinase 1	• Cell-cycle re-entry	0.02	[32]
ADORA1	Adenosine receptor A1	• Dysregulated excitatory synaptic transmission	0.01	[33]
AKR1C2	Aldo-keto reductase, family 1 member C2	• Anti-oxidation	0.01	[34]
ALOX12	Arachidonate 12-lipoxygenase	• Fatty acid metabolism<br>• Enhance oxidative stress	0.01	[35]
AVPR2	Arginine vasopressin receptor 2	• Blood brain barrier dysfunction<br>• Water-ion imbalance in the brain	0.01	[36]
CSNK2A1	Casein kinase II, alpha subunit	• Promote neuroinflammation	0.01	[37]
CXCR1	C-X-C motif chemokine receptor 1	• Microglia-specific neuroinflammation	0.01	[38]
MMP13	Matrix metalloproteinase 13	• Regulate Aβ production by BACE1	0.01	[39]
NOX4	NADPH oxidase 4	• Induce ferroptosis-dependent cytotoxicity in astrocytes<br>• Inhibit mitochondrial respiration in astrocytes	0.01	[40]
PIK3R1	Phosphatidyl inositol 3 kinase	• Inhibit GSK3β<br>• Suppress NF-κB transcription	0.01	[41]

References

[1] Lauretti E, Dincer O, Praticò D. Glycogen synthase kinase-3 signaling in Alzheimer's disease. Biochimica et Biophysica Acta (BBA) - Molecular Cell Research. 2020;1867(5):118664.

[2] Das B, Yan R. Role of BACE1 in Alzheimer's synaptic function. Transl Neurodegener. 2017;6:23-.

[3] Galle SA, van der Spek A, Drent ML, Brugts MP, Scherder EJA, Janssen JAMJL, et al. Revisiting the Role of Insulin-Like Growth Factor-I Receptor Stimulating Activity and the Apolipoprotein E in Alzheimer's Disease. Frontiers in Aging Neuroscience. 2019;11.

[4] Maki RA, Tyurin VA, Lyon RC, Hamilton RL, DeKosky ST, Kagan VE, et al. Aberrant Expression of Myeloperoxidase in Astrocytes Promotes Phospholipid Oxidation and Memory Deficits in a Mouse Model of Alzheimer Disease *. Journal of Biological Chemistry. 2009;284(5):3158-69.

[5] Arai T, Miklossy J, Klegeris A, Guo JP, McGeer PL. Thrombin and prothrombin are expressed by neurons and glial cells and accumulate in neurofibrillary tangles in Alzheimer disease brain. J Neuropathol Exp Neurol. 2006;65(1):19-25.

[6] Kuhnke D, Jedlitschky G, Grube M, Krohn M, Jucker M, Mosyagin I, et al. MDR1-P-Glycoprotein (ABCB1) Mediates Transport of Alzheimer's amyloid-beta peptides--implications for the mechanisms of Abeta clearance at the blood-brain barrier. Brain Pathol. 2007;17(4):347-53.

[7] Baloni P, Funk CC, Yan J, Yurkovich JT, Kueider-Paisley A, Nho K, et al. Metabolic Network Analysis Reveals Altered Bile Acid Synthesis and Metabolism in Alzheimer’s Disease. Cell Reports Medicine. 2020;1(8).

[8] Long H-Z, Cheng Y, Zhou Z-W, Luo H-Y, Wen D-D, Gao L-C. PI3K/AKT Signal Pathway: A Target of Natural Products in the Prevention and Treatment of Alzheimer's Disease and Parkinson's Disease. Frontiers in Pharmacology. 2021;12.

[9] Michael J, Unger MS, Poupardin R, Schernthaner P, Mrowetz H, Attems J, et al. Microglia depletion diminishes key elements of the leukotriene pathway in the brain of Alzheimer's Disease mice. Acta Neuropathologica Communications. 2020;8(1):129.

[10] Potapova TA, Daum JR, Byrd KS, Gorbsky GJ. Fine tuning the cell cycle: activation of the Cdk1 inhibitory phosphorylation pathway during mitotic exit. Mol Biol Cell. 2009;20(6):1737-48.

[11] Rosenfeld CS, Shay DA, Vieira-Potter VJ. Cognitive Effects of Aromatase and Possible Role in Memory Disorders. Frontiers in Endocrinology. 2018;9.

[12] Singh P, Ravanan P, Talwar P. Death Associated Protein Kinase 1 (DAPK1): A Regulator of Apoptosis and Autophagy. Frontiers in Molecular Neuroscience. 2016;9.

[13] Quartey MO, Nyarko JNK, Pennington PR, Heistad RM, Klassen PC, Baker GB, et al. Alzheimer Disease and Selected Risk Factors Disrupt a Co-regulation of Monoamine Oxidase-A/B in the Hippocampus, but Not in the Cortex. Frontiers in Neuroscience. 2018;12.

[14] Fragkouli A, Tsilibary EC, Tzinia AK. Neuroprotective role of MMP-9 overexpression in the brain of Alzheimer's 5xFAD mice. Neurobiology of Disease. 2014;70:179-89.

[15] Cacabelos R, Cacabelos P, Torrellas C. Personalized Medicine of Alzheimer's Disease. Handbook of Pharmacogenomics and Stratified Medicine. 2014:563-615.

[16] Murakami M, Sato H, Miki Y, Yamamoto K, Taketomi Y. A new era of secreted phospholipase A2. Journal of Lipid Research. 2015;56(7):1248-61.

[17] Tai LM, Loughlin AJ, Male DK, Romero IA. P-Glycoprotein and Breast Cancer Resistance Protein Restrict Apical-to-Basolateral Permeability of Human Brain Endothelium to Amyloid-β. Journal of Cerebral Blood Flow & Metabolism. 2009;29(6):1079-83.
[18] Shen S, Callaghan D, Juzwik C, Xiong H, Huang P, Zhang W. ABCG2 reduces ROS-mediated toxicity and inflammation: a potential role in Alzheimer's disease. Journal of Neurochemistry. 2010;114(6):1590-604.
[19] Wang X-X, Tan M-S, Yu J-T, Tan L. Matrix Metalloproteinases and Their Multiple Roles in Alzheimer's Disease. BioMed Research International. 2014;2014:908636.
[20] Pentz R, Iulita MF, Mikutra-Cencora M, Ducatenzeiler A, Bennett DA, Cuello AC. A new role for matrix metalloproteinase-3 in the NGF metabolic pathway: Proteolysis of mature NGF and sex-specific differences in the continuum of Alzheimer's pathology. Neurobiology of Disease. 2021;148:105150.
[21] Wang L, Chiang H-C, Wu W, Liang B, Xie Z, Yao X, et al. Epidermal growth factor receptor is a preferred target for treating Amyloid-β–induced memory loss. Proceedings of the National Academy of Sciences. 2012;109(41):16743-8.
[22] Terni B, Ferrer I. Abnormal Expression and Distribution of MMP2 at Initial Stages of Alzheimer's Disease-Related Pathology. Journal of Alzheimer's Disease. 2015;46:461-9.
[23] Horgusluoglu-Moloch E, Risacher SL, Crane PK, Hibar D, Thompson PM, Saykin AJ, et al. Genome-wide association analysis of hippocampal volume identifies enrichment of neurogenesis-related pathways. Scientific Reports. 2019;9(1):14498.
[24] Shalini S-M, Ho CF-Y, Ng Y-K, Tong J-X, Ong E-S, Herr DR, et al. Distribution of Alox15 in the Rat Brain and Its Role in Prefrontal Cortical Resolvin D1 Formation and Spatial Working Memory. Molecular Neurobiology. 2018;55(2):1537-50.
[25] Butler PM, Chiong W, Perry DC, Miller ZA, Gennatas ED, Brown JA, et al. Dopamine receptor D(4) (DRD(4)) polymorphisms with reduced functional potency intensify atrophy in syndrome-specific sites of frontotemporal dementia. Neuroimage Clin. 2019;23:101822-.
[26] Barski OA, Tipparaju SM, Bhatnagar A. The aldo-keto reductase superfamily and its role in drug metabolism and detoxification. Drug Metab Rev. 2008;40(4):553-624.
[27] Chen F, Wollmer MA, Hoerndli F, Münch G, Kuhla B, Rogaev EI, et al. Role for glyoxalase I in Alzheimer's disease. Proc Natl Acad Sci U S A. 2004;101(20):7687-92.
[28] Krohn M, Lange C, Hofrichter J, Scheffler K, Stenzel J, Steffen J, et al. Cerebral amyloid-β proteostasis is regulated by the membrane transport protein ABCC1 in mice. J Clin Invest. 2011;121(10):3924-31.
[29] Jang BG, Yun S-M, Ahn K, Song JH, Jo SA, Kim Y-Y, et al. Plasma Carbonic Anhydrase II protein is Elevated in Alzheimer's Disease. Journal of Alzheimer's Disease. 2010;21:939-45.
[30] Cho S-J, Park MH, Han C, Yoon K, Koh YH. VEGFR2 alteration in Alzheimer's disease. Scientific Reports. 2017;7(1):17713.
[31] Hamasaki H, Honda H, Suzuki SO, Hokama M, Kiyohara Y, Nakabeppu Y, et al. Down-regulation of MET in hippocampal neurons of Alzheimer's disease brains. Neuropathology. 2014;34(3):284-90.
[32] Song B, Davis K, Liu XS, Lee H-g, Smith M, Liu X. Inhibition of Polo-like kinase 1 reduces beta-amyloid-induced neuronal cell death in Alzheimer's disease. Aging (Albany NY). 2011;3(9):846-51.
[33] Hohoff C, Garibotto V, Elmenhorst D, Baffa A, Kroll T, Hoffmann A, et al. Association of Adenosine Receptor Gene Polymorphisms and In Vivo Adenosine A1 Receptor Binding in The Human Brain. Neuropsychopharmacology. 2014;39(13):2989-99.
[34] Turkez H, Cacciatore I, Arslan ME, Fornasari E, Marinelli L, Di Stefano A, et al. Histidyl-Proline Diketopiperazine Isomers as Multipotent Anti-Alzheimer Drug Candidates. Biomolecules. 2020;10(5):737.
[35] Praticò D, Zhukareva V, Yao Y, Uryu K, Funk CD, Lawson JA, et al. 12/15-lipoxygenase is increased in Alzheimer's disease: possible involvement in brain oxidative stress. Am J Pathol. 2004;164(5):1655-62.
[36] Viñuela-Berni V, Gomez-Gonzalez B, Quintanar-Stephano A. Blockade of Arginine Vasopressin receptors prevents blood-brain barrier breakdown in Experimental Autoimmune Encephalomyelitis. Scientific Reports. 2020;10:467.
[37] Rosenberger AFN, Morrema THJ, Gerritsen WH, van Haastert ES, Snkhchyan H, Hilhorst R, et al. Increased occurrence of protein kinase CK2 in astrocytes in Alzheimer's disease pathology. J Neuroinflammation. 2016;13:4-.
[38] Liu C, Cui G, Zhu M, Kang X, Guo H. Neuroinflammation in Alzheimer's disease: chemokines produced by astrocytes and chemokine receptors. Int J Clin Exp Pathol. 2014;7(12):8342-55.
[39] Zhu BL, Long Y, Luo W, Yan Z, Lai YJ, Zhao LG, et al. MMP13 inhibition rescues cognitive decline in Alzheimer transgenic mice via BACE1 regulation. Brain. 2019;142(1):176-92.
[40] Park MW, Cha HW, Kim J, Kim JH, Yang H, Yoon S, et al. NOX4 promotes ferroptosis of astrocytes by oxidative stress-induced lipid peroxidation via the impairment of mitochondrial metabolism in Alzheimer's diseases. Redox Biol. 2021;41:101947-.
[41] Gabbouj S, Ryhänen S, Marttinen M, Wittrahm R, Takalo M, Kemppainen S, et al. Altered Insulin Signaling in Alzheimer's Disease Brain – Special Emphasis on PI3K-Akt Pathway. Frontiers in Neuroscience. 2019;13.
